# Electromagnetic Sensor-Guided Enteral Access Systems: A Literature Review

**DOI:** 10.1007/s00455-015-9607-4

**Published:** 2015-05-06

**Authors:** David Smithard, Nicholas A. Barrett, David Hargroves, Stuart Elliot

**Affiliations:** King’s College, London, UK; Guy’s and St Thomas’ NHS Foundation Trust, London, UK; East Kent Hospitals University NHS Foundation Trust, Kent, UK; Leeds Teaching Hospitals NHS Trust, Leeds, England

**Keywords:** Enteral feeding, Electromagnetic, Nasogastric tube

## Abstract

Enteral feeding is the nutritional support of choice for acutely ill patients with functional gastrointestinal tracts who are unable to swallow. Several benefits including reduced mortality and length of hospital stay have been associated with early initiation of enteral feeding. However, misplacement of conventional nasoenteric tubes is relatively common and can result in complications including pneumothorax. In addition, the need to confirm the position by X-ray can delay the start of using the tube. Eliminating these delays can help patients start feeding, and minimise the adverse impact on initiating hydration and medication. The purpose of this review was to critically examine whether electromagnetic sensor-guided enteral access systems (EMS-EAS) can help overcome the challenges of conventional nasoenteric feeding tube placement and confirmation. The Royal Society of Medicine’s library performed two searches on Medline (1946–March 2014) and Embase (1947–March 2014) covering all papers on Cortrak or electromagnetic or magnetic guidance systems for feeding tubes in adults. Results from the literature search found an agreement between the radiographic and EMS-EAS confirmation of placement. EMS-EAS virtually eliminated the risk of misplacement and pneumothorax was not reported. In addition, studies showed a small decrease in the number of X-rays with EMS-EAS and a reduced average time to start feeding compared with blind placement. This review suggests that EMS-EAS reduces several complications associated with the misplacement of nasoenteric feeding tubes, and that there could be considerable improvements in mortality, morbidity, patient experience and cost if EMS-EAS is used instead of conventional methods.

## Introduction

John Hunter made the first reported attempts at nasogastric (NG) or orogastric feeding in 1769 [1]. However, the technique was used infrequently until Dobbie and Hoffmeister reported successful outcomes with small-bore, weighted tubes in 1976 [1]. Today, enteral feeding is the nutritional support of choice for acutely ill medical and critical care patients with functional gastrointestinal tracts who are unable to swallow [2–4].

Although the optimal time to start enteral nutrition is uncertain, “early” initiation appears to have significant advantages. Trials that enrolled critically ill patients demonstrated several benefits associated with enteral nutrition including improvements in nitrogen balance, splanchnic blood flow, gastrointestinal mucosal barrier function, mortality among mechanically ventilated patients and length of hospital stay [2, 3, 5, 6]. Evidence is less clear in patients suffering with an acute neurovascular event; protein-energy malnutrition during the first week after an acute stroke increased the risk of death or Barthel index ≤50 on the 30th day of follow-up 3.5-fold, whereas, the FOOD study, although suggesting a modest absolute risk reduction in mortality and poor functional outcome, was not statistically significant (1.2 %, −4.2 to 6.6, *p* = 0.7) [7, 8].

Misplacement of conventional nasoenteric (NG or nasojejunal [NJ]) tubes is relatively common and can result in significant complications [9, 10]. Between September 2005 and March 2011, the National Patient Safety Agency (NPSA) in the United Kingdom (UK) received reports of 21 deaths and 79 cases of harm related to feeding through NG tubes misplaced into the lower bronchial tree rather than the enteral tract.[9]. Due to the voluntary reporting of these adverse incidents and the reporting of misplaced tubes only where harm has occurred, the NPSA figures may underestimate the true incidence. In addition, misplaced tubes are frequently repositioned before use and therefore not reported as an incident. Indeed, numerous studies allude to the underreporting of nasoenteric tube misplacement in a variety of settings [3, 10–12].

Other significant harm associated with nasoenteric tube insertion and misplacement include pneumothorax [10], vocal cord injury (NG tube syndrome), bronchopleural fistula, aspiration pneumonia with or without emphysema, perforation of the membranous trachea or pleural parenchyma, hydrothorax, mediastinitis, atelectasis and plural effusions [5, 13]. The true incidence of these complications is unknown.

Several patient-related factors increase the risk of nasoenteral tube misplacement including tracheal intubation and mechanical ventilation, depressed levels of consciousness (regardless of cause), vocal cord dysfunction and swallowing dysfunction [9, 13]. In addition, a reduced reflex or impaired gag reflex may contribute to poor recognition of a misplaced NG tube. Unfortunately, patients most likely to need enteral feeding often have one or more factors that predispose to misplacement.

### Techniques to Minimise the Risk of Misplacement

National Health Service (NHS) England specifies that healthcare professionals should measure the pH of an aspirate of approximately 1–2 ml of the gastric contents to confirm enteral placement. A pH of 1–5.5 confirms the tube is in the stomach [9]. However, acid pH might be recorded in the oesophagus in patients with conditions such as hiatus hernia and gastro-oesophageal reflux. Other patients, such as those taking proton pump inhibitors or requiring continuous enteral feeds, will have a neutral or alkaline gastric pH [3]. A UK study reported obtaining gastric aspirates in 60 % of 43 post-pyloric feeding tube placements that were suitable for pH readings. The pH was ≤5 in 44 % (19/43) of the placements [14]. Another study reported that a pH < 5.0 confirmed the gastric placement of 60 % of tubes [10]. Difficulty obtaining an aspirate may delay the start of using the tube for feeding, hydration or medication.

NHS England recommends obtaining a chest radiograph if the pH test does not confirm the correct placement of nasoenteric tubes [9]. However, radiological misinterpretation is the most common cause of severe harm incidents associated with nasoenteric tubes reported to the NPSA. Twelve of the 45 incidents associated with radiological misinterpretation resulted in fatalities [9].

Bronchial intubation may cause pulmonary trauma between placement and radiological confirmation of the inappropriate position [10, 15]. Radiographs are only accurate at the time they are taken and additional radiographs may be required if the nasoenteric tube is clinically suspected of moving from the initial placement following, for example, coughing, retching or vomiting. For instance, between 27 and 42 % of NJ tubes show retrograde migration into the duodenum or stomach [16], while NG tubes can move to the oesophagus or post-pylorically. Repeated X-ray exposure carries a small, but appreciable, carcinogenic risk. For example, in the UK, diagnostic X-rays account for about 0.6 % of the cumulative risk of cancer before the age of 75 years, equivalent to about 700 cases a year [17].

Furthermore, transfer to the radiology department, along with the production, interpretation and reporting of X-rays potentially delays the start of feeding, hydration and medication. Eliminating these delays helps patients start tube feeding more rapidly, thereby increasing the proportion that attain their caloric and nutrient targets, and minimising delays to the start of hydration and medication [18].

This review examines whether the electromagnetic sensor-guided enteral access system (hereafter EMS-EAS)—of which CORTRAK™ (CORPAK MedSystems UK, Gatwick, UK) is the only example on the UK market—helps overcome the challenges of conventional nasoenteric feeding tube placement and confirmation. EMS-EAS, a bedside system, uses an electromagnetic sensor to track and display the anterior and cross-sectional path of a polyurethane feeding tube and transmitting stylet assembly during NG or post-pyloric placement [13].

## Methods

The Royal Society of Medicine’s library performed two searches on Medline (1946–March 2014) and Embase (1947–March 2014) covering

- All papers on Cortrak or electromagnetic or magnetic guidance systems for feeding tubes in adults. The search excluded blind placement, endoscopic placement and studies on animals or children.
- Cost-effectiveness or safety of blind placement of feeding tubes—excluding endoscopic placement and studies in animals or children.

The search was restricted to studies published in English. Corpak MedSystems provided selected information from meetings, which we augmented with further searches of congress websites. Reference lists were manually searched to include additional references identified in these searches and excluded, as far as possible, duplicate studies. Appendix one shows the search strategies. All authors reviewed the results of the literature searches to ensure that all relevant publications were included.

## Results

### Accuracy of Placement With EMS-EAS Compared to X-Ray

Several studies compared gastric or post-pyloric (duodenum or jejunum) position indicated with EMS-EAS with that shown on radiographs (Table 1). These studies indicated an agreement between the radiographic and EMS-EAS confirmation of the tubes.

**Table 1:** Radiologically confirmed placements of nasogastric tubes using EMS-EAS

Patients recruited to the ICU (n)	Mean age (years)	Diagnosis category (%)	Number of radiologically confirmed placements	Total number of placements		Percentage of radiologically confirmed placements (%)	Reference
25	NA	NA	25	25		100.0	Ackerman et al. [28]
74	67 ± 19	Medical, 73 Surgery, 24 Trauma, 3	61	74		82.4	Boyer et al. [39]
52	NA	NA	57	57		100.0	Lei et al. [20]
25	NA	NA	24	24		100.0	Phang et al. [40]
194 (18 paediatric patients) ICU 78.4 % non-ICU 12.4 % Paediatric 9.2 %	55 ± 22	Medical, 50.2 Neurological, 25.4 Trauma, 13.2 Surgery, 11.2	193	194		99.5	Powers et al. [41]
27	NA	NA	20	21		95.2	Priestley et al. [42]
NA	NA	NA	470	483		97.3	Stockdale et al. [43]
113	Median, 53 (IQR, 36, 66)	Medical, 30 Neurological, 12 Trauma, 44 Surgery, 14	127	127		100.0	Taylor et al. [10]
142	NA	NA	135	135		100.0	Wang et al. [38]
			Totals				
			1112	1140		Mean = 97.5 %	

*ICU* intensive care unit, *IQR* interquartile range, *NA* not available

### Bronchial Misplacement

Tables 2 and 3 show the number of nasoenteric tubes misplaced in the bronchi in studies of conventional placement and EMS-EAS and the number of misplacements avoided (i.e. where EMS-EAS detected entry into the upper airway allowing the tube to be repositioned before final placement). The number of nasoenteric tubes misplaced in the bronchi indicates that EMS-EAS virtually eliminates the risk of misplacement.

**Table 2:** The number of nasoenteric tubes misplaced in the bronchi with conventional placement

Patients recruited to the ICU, n	Mean age, years	Pulmonary placements	Total placements	Reference
Comparative studies				
729 ICU 65.7 % Non-ICU 34.3 %	Median, 59 (18–98)	27	1822	Aguilar-Nascimento and Kudsk [15]
214	5 (18–101)	2	242	Hillard et al. [25]
ICU and medical surgical unit, 101	61	3	101	McCutcheon et al. [27]
Non-comparative studies				
4190	NA	108	5158	Marderstein et al. [44]
NA	NA	14	1100	McWey et al. [45]
740	NA	14	740	Rassias et al. [46]
NA	71 (22–91)	50	3789	Sorokin et al. [11]
Medical and surgical ICU Inpatient acute care	NA	187	9931	Sparks et al. [3]
NA	NA	1	43	Gatt et al. [14]
		Totals (%)		
		406 (1.77)	22926	

*ICU* intensive care unit

**Table 3:** The number of nasoenteric tubes misplaced in the bronchi and the number of misplacements avoided with EMS-EAS

Patients recruited to the ICU, n	Mean age, years	Diagnosis category, %	Pulmonary placements	Total placements	Misplacements avoided (%)^a^	Total placements	Reference
Comparative studies							
ICU and medical surgical unit, 84	54	NA	0	84			McCutcheon et al. [27]
Non-comparative studies							
715	58 ± 18	NA	0	1154			Koopman et al. [34]
194 (18 paediatric patients) ICU 78.4 % non-ICU 12.4 % Paediatric 9.2 %	55 ± 22	Medical, 50.2 Neurological, 25.4 Trauma, 13.2 Surgery, 11.2	0	194	15	194	Powers et al. [41]
632	63 ± 15	Cardiovascular unit, 23 Medical, 48 TSN, 29	0	904			Powers et al. [47]
616	63 ± 16	Cardiac, 30.3 Medical, 13.4 Neurological, 19.9 Non-ICU, 10.3 Surgery, 18.2 Vascular, 7.9	0	719	“on occasion”	719	Rivera et al. [48]
NA	NA	NA	0	483			Stockdale et al. [43]
	Median, 44	Medical, 21 % Neurosurgical, 9 Surgical, 21 Trauma, 49	0	799	26	799	Taylor et al. [30]
200	65 (1–16)	NA	0	200			Trottier et al. [49]
25	NA	NA			4	25	Ackerman et al. [28]
20	NA	Cardiothoracic General	0	20			Lee et al. [6]
142	NA	NA	0	142	2	142	Wang et al. [38]
			Totals (%)				
			0 (0)	4699	47 (4.05)	1160	

*ICU* intensive care unit, *NA* not available, *TSN* trauma/surgical/neurological unit

^a^Attempts where the tube entered the bronchi, but EMS-EAS detected the misplacement allowing the tube to be repositioned before final placement

The literature search identified a single report of a serious incident arising from unrecognised intra-bronchial placement using EMS-EAS [19]. CORPAK MedSystems received four such reports in the UK since launch in 2005 (Corpak Personal communication. 2014). To place these results in context, between January 2010 and April 2014, CORPAK MedSystems sold approximately 17,700 EMS-EAS tubes in the UK alone (Corpak Personal communication. 2014).

### Delay in the start of tube feeding

Based on studies that enrolled patients requiring post-pyloric tubes, the mean of the average time to start of enteral feeding was 21.5 h with blind placement and 11.5 using EMS-EAS (Table 4).

**Table 4:** Time to start enteral nutrition with blind and EMS-EAS-guided placement of post-pyloric tubes

Blind placement (h)	EMS-EAS (h)	Reference
Comparative studies		
22.3	7.8	Gray et al. [5]
28.6	19.7	MacKay et al. [24]
22.7	7.0	McCutcheon et al. [27]
Non-comparative studies		
6 (IQR 5–18)		Gatt et al. [14]
28.1		Hillard et al. [25]
Mean of averages		
21.5	11.5	

*IQR* interquartile range

### Radiological Exposure

The number of X-rays received was similar between patients receiving a nasoenteric tube with blind placement (mean of averages 2.11) and EMS-EAS (mean of averages 1.22, Table 5).

**Table 5:** Number of X-rays required to confirm tube position with blind placement and EMS-EAS

Blind placement	EMS-EAS placement	Reference
Comparative studies		
2	1	Gray et al. [5]
1.49	1.13	Koopman et al. [34]
1.55	1.45	MacKay et al. [24]
3.40	1.02	McCutcheon et al. [27]
Non-comparative studies		
	1.5	Aguilar-Nascimento and Kudsk [15]
2.1		Hillard et al. [25]
Mean of the averages		
2.11	1.22	

### Placement Time

Only one study directly compares the time to confirmed placement of a NG tube using pH monitoring with EMS-EAS (Table 5): 11.6 and 9.6 min, respectively [20]. Blind placement of a post-pyloric tube takes, on average, 42 min compared with 15.5 min using EMS-EAS (mean of averages)(Table 6).

**Table 6:** Time needed for conventional placement of feeding tubes and placement guided by EMS-EAS

Blind placement (min)	EMS-EAS (min)	Reference
NG tubes		
11.6 (SE ± 1.7)^a^	9.6 (SE ± 1.7)	Lei et al. [20]
	0.48 (IQR^d^ 0.34–1.09)	Roa et al. [2]
	9 (IQR 6–14)^b^	Taylor et al. [33]
	6.4 (IQR 4–10.4)	Taylor et al. [10]
Mean of averages		
11.6	6.4	
Post-pyloric tubes		
60	10	Phang et al. [40]
37	12.5	Stockdale et al. [22]
28 (10-90)		Cresci et al. [50]
	5.9^c^	Deane et al. [37]
	30	Dolan et al. [21]
	12.4	Duflou et al. [36]
	18 (IQR 14–30)	Gatt et al. [14]
	11 (IQR 6–19)	Holzinger et al. [51]
	7.6 (range 1–20)	Kaffarnik et al. [52]
	18 (range 3-55)	Lee et al [6]
	16.3 (SD ± 11.8)	Mathus-Vliegen et al. [53]
	14.8 (SD ± 14.7)	
	26.2 (SD ± 19.3)	
	12 (range 1–52)	Powers et al. [41]
	6.16 (IQR^d^ 3.55–9.03)	Roa et al. [2]
	30 ± 17	Trottier et al. [49]
	12.6 (range^d^ 5.3–34.4)	Young et al. [54]
	20.12 (SD ± 3.71)	Wang et al. [38]
Mean of averages		
42	15.5	

*IQR* interquartile range, *SD* standard deviation

^a^Based on pH paper

^b^Last 20 patients to allow for training effect

^c^Last 50 patients to allow for training effect

^d^unclear from paper

### Pneumothorax

A reduction in the incidence of pneumothorax and iatrogenic pneumothorax has been seen with EMS-EAS in the studies to date (Table 7). CORPAK MedSystems have received no reports of pneumothorax in the UK between the launch of the EMS-EAS in 2005 and April 2014.

**Table 7:** Number of iatrogenic pneumothoraces following blind- and EMS-EAS-guided placement

Blind placement			EMS-EAS			Reference
Cases	Number of patients	% (range)	Cases	Number of patients	% (range)	
Comparative studies						
11	831	1.32	0	715	0	Koopman et al. [34]
1	101	0.99	0	84	0	McCutcheon et al. [27]
Non-comparative studies						
9	729	1.23				Aguilar-Nascimento and Kudsk [15]
9	4190	0.21				Marderstein et al. [44]
4	1100	0.36				McWey et al. [45]
5	740	0.68				Rassias et al. [46]
8	2079	0.38				Sorokin et al. [11]
			0	194	0	Powers et al. [41]
			0	616	0	Rivera et al.
			0	483	0	Stockdale et al. [22]
			0	69	0	Taylor et al. [30]
			0	142	0	Wang et al. [38]
Total						
47	9770	0.48	0	2303	0	

## Discussion

Early enteral nutrition in acutely ill patients appears to reduce mortality and morbidity [2, 3, 5–7]. Nasoenteric feeding has a recognised morbidity and mortality associated with misplacement of the tube into the bronchial tree [9, 10]. This review of the literature of EMS-EAS compared with blind placement suggests that EMS-EAS can reduce the risk of feeding into the lungs, pneumothorax and time to commence feeding. More rapid and safer tube insertion reduces morbidity and is cost effective compared to blind placement and fluoroscopy using a variety of estimates, settings, countries and outcomes [5, 18, 20–27].

The position of the tube on EMS-EAS and X-ray agreed in 98 % of cases. It is unclear why in 2 % of cases there was a difference, however, potential reasons include tube migration between the NG insertion and radiological confirmation, operator error in positioning the tube using EMS-EAS, patient anatomy and incorrect interpretation of the X-ray. Nevertheless, given the apparent high level of confirmation between the enteral feeding tube tip position using EMS-EAS and X-ray, it seems reasonable that EMS-EAS could replace radiological confirmation of the nasoenteric tube’s position for most patients [28]. This avoids the potential damage to the respiratory tree that might occur given the delay between misplacement and radiography. Moreover, Sparks et al reported that between 13 and 32 % of subsequent blind intubations were incorrectly positioned [3]. EMS-EAS eliminates “the cost and patient safety burden of [these] additional X-rays” [28].

Inadvertent placement into the bronchi occurs in 2–4 % of blind insertions of nasoenteric tubes. Differences in patient population, sample size, reporting bias and the method of identifying tube misplacement might contribute to variations in the incidence of pulmonary placement of feeding tubes. Up to 80 % of these misplacements are not clinically detected [13] and require routine X-ray detection [1]. The potential for serious, but avoidable, complications is considerable. The NHS used approximately 271,000 nasoenteric tubes during 2008 [29]. Assuming that 2–4 % of nasoenteric tubes inserted with conventional placement enter the pulmonary system, there are approximately 5,000–110,00 misplaced tubes per annum, all of which have the potential to cause significant morbidity and mortality.

The literature suggests a rate of pneumothorax from 18.7–26 % of bronchial tube placements with an associated mortality of 2.7–4 % [3]. Sparks et al, for example, reported that 18.7 % of the nasoenteric tubes misplaced into the bronchial tree resulted in pneumothoraces, while 2.7 % were fatal [3]. Sorokin et al reported that 26 % of patients with a misplaced tube experienced pneumothoraces and other complications, with a mortality rate directly attributed to the misplacement of 4 % [11]. The present review demonstrated a significant reduction in pneumothorax associated with EMS-EAS use with a single report of a serious incident arising from unrecognised intra-bronchial placement using EMS-EAS [19]. The reduction with EMS-EAS is likely to be because, unlike X-ray, EMS-EAS detects in real-time when a nasoenteric tube enters the upper reaches of the bronchial tree allowing the healthcare professional to reposition the tube before final placement [30].

These figures are considerably higher than the mortality reported to the NPSA: 21 deaths between September 2005 and March 2011 [9]. This may suggest there is under-reporting of harm caused by misplaced nasoenteric tubes, possibly caused by misattribution of mortality to co-morbidities in this severely ill population. Numerous studies indicate underreporting of adverse events associated with pharmaceuticals through spontaneous reports. [31, 32] There seems to be no reason why spontaneous reports would not also under-represent adverse events associated with devices. Indeed, many authors comment that healthcare professionals probably underestimate the prevalence of, and risks associated with, misplaced nasoenteric tubes [3, 10–12]. In the study by Sorokin et al, a search of radiology reports identified misplacements. In contrast, their risk management database did not include any of the misplacements [11]. Indeed, some commentators report that they know of cases that were missed by the search for misplacement. [1] Clearly, there is a pressing need to improve reporting of these potentially fatal adverse events.

Minimising the delay to the start of enteral feeding helps improve outcomes in critically ill patients [2, 3, 5]. Several studies suggest that EMS-EAS allows earlier initiation of enteral feeding, probably through a combination of more rapid intubation as well as by avoiding X-rays. Similarly, the median proportion of critically ill people with delayed gastric emptying that attain the enteral nutrition goal increased from 19 % with conventional NJ tube placement to between 80 and 100 % following EMS-EAS’s implementation [33]. The reduction in time to start feeding is consistent across the literature [5, 15, 24, 25, 27, 34]. A recent intensive care unit study reported that EMS-EAS confirmed placement of NG tubes took a mean of 9.6 minutes (standard error [SE] ± 1.7), while patients who required an X-ray took 122 (SE ± 23; *p* < 0.0001) minutes [20], equivalent to a 92 % reduction if EMS-EAS were used instead of X-ray confirmation. In this study, the time to feeding was 3.98 h with conventional placement of NG tubes compared to 2.58 h using EMS-EAS (*p* = 0.049) [20]. The present review suggests that healthcare professionals can insert NG and post-pyloric tubes more rapidly using EMS-EAS than conventional placement (Table 5), although times vary considerably. The delay between intubation and X-ray depends on numerous factors including the distance between the ward and the radiological suite as well service provision, such as operator experience, operator training and limited numbers of radiographers over weekends and public holidays.

In the UK, NHS England guidance recommends pH testing and X-ray testing only if the position is not confirmed. The use of pH may confirm that the tip is in an acidic environment, but does not confirm subdiaphragmatic placement as the patient may have a hiatus hernia or reflux disease—the prevalence of gastro-oesophageal reflux in Western Europe is estimated to lie between 8.8 and 25 % [35]. Taylor et al [10] reported that pH test of <5.0 confirmed gastric placement in only 60 % of tubes. Another UK study reported obtaining gastric aspirates in 60 % of 43 intubations and a pH reading of ≤5 in 44 % [14]. Therefore, it is estimated that in the UK 40 % of patients with an NG placement (approximately 110,000) will need an X-ray following failure to measure pH. This use of X-rays introduces a burden of radiation for patients as well as a significant cost (the cost of a conventional X-ray in the NHS is approximately £25, the 110,000 X-rays cost the UK taxpayer £2.7 million annually).

### Limitations and Future Research

This review is subject to several limitations that are common to literature reviews. There were no prospective randomised controlled trials reported in the literature and all studies were cohort or case-control studies. Methodological uncertainties (for example, whether the investigators used the same start and end points when assessing timings, and differences in service settings and protocols) can complicate interpretation of these data. Furthermore, the literature was predominantly from the USA and UK where different protocols are followed that may influence aspects such as the need for X-ray confirmation following tube placement.

Many studies are posters or available only as abstracts and there appears to be an overlap in some of the published cohorts, although we endeavoured, as far as possible, to exclude potential duplicates. The studies came from diverse settings, enrolled diverse cohorts and employed diverse methodologies. These differences and the level of detail presented in the posters and papers precluded a meta-analysis, which was our original intention. This highlights the need for formal prospective studies ideally in a single setting (e.g. stroke units and a defined patient cohort from the intensive care unit).

The true costs of an X-ray are dependent upon the healthcare setting in which the X-ray are taken. Relatively few studies ascertain the cost-effectiveness of EMS-EAS from the perspective of the NHS. A study from St Thomas’ Hospital suggested that using EMS-EAS for 57 insertions in 52 patients requiring NG placement potentially avoided 46 chest X-rays, which equated to a saving of £2300. The costs estimated in this study are from one author’s institution and represent inter-departmental cross charging rather than an absolute cost (This study estimated that an X-ray cost £50.) [20] Costs are therefore likely to be underestimated and do not include indirect costs such as those associated with treating cancers caused by X-rays, the consequences of delayed nutritional support, hydration or medication, and the opportunity costs associated when healthcare professionals accompany patients to X-ray. Clearly, there is a need for further economic studies encompassing the range of costs and consequences associated with conventional placement and EMS-EAS.

Finally, it is possible that the effect of EMS-EAS may be overestimated. Centres that participate in clinical studies may be more experienced and, therefore, less likely to cause adverse events than might be expected in general clinical practice. Several studies report a learning curve or comment that experience enhances the success of EMS-EAS and nastoenteric tube placement [3, 14, 33, 36–38]. For example, Deane et al reported that the time to place a post-pyloric tube declined from 20.8 min in the first 10 patients to 5.9 min in the next 50 placements (*p* = 0.003) which underlines the importance of training [37]. Future studies should address this.

## Conclusions

This literature review of the use of EMS-EAS and blind placement suggests that there is a *prima facie* case that EMS-EAS reduces the risk of bronchial misplacement of feeding tubes, pneumothorax, time to commence feeding and, presumably, other complications associated with the misplacement of nasoenteric feeding tubes. This suggests that there are considerable improvements in mortality, morbidity, patient experience and cost if EMS-EAS is used instead of conventional methods to confirm NG position. Further prospective studies and analyses need to confirm the findings in this review.

## Appendix: Search Strategies

Summary of search: All papers on Cortrak or electromagnetic/magnetic guidance systems for feeding tubes—excluding blind placement, endoscopic placement and studies on animals or children by manually scanning the final results.

**Table:**

Set#	Searched for	Databases	Results
S11	s8 or s10	Embase®, Embase® Alert, MEDLINE®	197^a^
S10	(s9 not (s8 or “magnetic resonance” or “magnet [6^a^] endoscop [6^a^]” or mei or mri or mris)) and la (english)	Embase®, Embase® Alert, MEDLINE®	124
S9	magnet [6^a^] and (s3 or s4)	Embase®, Embase® Alert, MEDLINE®	1356
S8	(s1 or s2 or s7) and la (english)	Embase®, Embase® Alert, MEDLINE®	78
S7	(s3 or s4) and (s5 or s6)	Embase®, Embase® Alert, MEDLINE®	84
S6	electromagnet [6^a^] or “electro magnet [6^a^]”	Embase®, Embase® Alert, MEDLINE®	71272
S5	MESH.EXACT (“Electromagnetic Fields”) OR MESH.EXACT (“Electromagnetic Phenomena”) OR EMB.EXACT (“electromagnetic radiation”) OR EMB.EXACT (“electromagnetic field”)	Embase®, Embase® Alert, MEDLINE®	38915
S4	(Enteral [2^a^] or enteric or post-pyloric or pyloric or nasointestinal or intestinal or nasojejunal or jejunal or nasogastric or gastric or gastrointestinal or gi or orogastric or nasoduodenal or duodenal or intraintestinal or intragastric or nasoenteral [2^a^] or nasoenteric or nose or nasal or feeding) near/5 (tube [1^a^] or device [1^a^] or catheter [1^a^] or intubat [4^a^])	Embase®, Embase® Alert, MEDLINE®	62430
S3	MESH.EXACT (“Enteral Nutrition”) OR MESH.EXACT (“Intubation, Gastrointestinal”) OR EMB.EXACT (“enteric feeding”) OR EMB.EXACT (“nose feeding”) OR EMB.EXACT (“feeding apparatus”) OR EMB.EXACT.EXPLODE (“digestive tract intubation”) OR EMB.EXACT.EXPLODE (“nasogastric tube”) OR EMB.EXACT (“stomach tube”)	Embase®, Embase® Alert, MEDLINE®	60610
S2	“enteral access system” or egnt	Embase®, Embase® Alert, MEDLINE®	9
S1	Cortrak	Embase®, Embase® Alert, MEDLINE®	24

^a^The search strategy retrieved a number of references that were then manually searched to find the most relevant

Cost-effectiveness or safety of blind placement of feeding tubes—excluding endoscopic placement, studies on animals or children, and a small number of references duplicated in the Cortrak search by manually scanning the final results.

**Table:**

Set#	Searched for	Databases	Results
S3	(s1 or s2) and blind [2^a^] and (place [1^a^] or placing or placement [1^a^] or insert [4^a^] or passage [1^a^]) and la (english)	Embase®, Embase® Alert, MEDLINE®	404^a^
S2	(enteral [2^a^] or enteric or post-pyloric or pyloric or nasointestinal or intestinal or nasojejunal or jejunal or nasogastric or gastric or orogastric or gastrointestinal or gi or nasoduodenal or duodenal or intraintestinal or intragastric or nasoenteral [2^a^] or nasoenteric or nose or nasal or feeding) near/5 (tube [1^a^] or device [1^a^] or catheter [1^a^] or intuba [4^a^])	Embase®, Embase® Alert, MEDLINE®	62430
S1	MESH.EXACT (“Enteral Nutrition”) OR MESH.EXACT (“Intubation, Gastrointestinal”) OR EMB.EXACT (“enteric feeding”) OR EMB.EXACT (“nose feeding”) OR EMB.EXACT (“feeding apparatus”) OR EMB.EXACT.EXPLODE (“digestive tract intubation”) OR EMB.EXACT.EXPLODE(“nasogastric tube”) OR EMB.EXACT (“stomach tube”)	Embase®, Embase® Alert, MEDLINE®	60610

^a^The search strategy retrieved a number of references that were then manually searched to find the most relevant
